# RAB-5 Controls the Cortical Organization and Dynamics of PAR Proteins to Maintain *C. elegans* Early Embryonic Polarity

**DOI:** 10.1371/journal.pone.0035286

**Published:** 2012-04-24

**Authors:** Vincent Hyenne, Thierry Tremblay-Boudreault, Ramraj Velmurugan, Barth D. Grant, Dinah Loerke, Jean-Claude Labbé

**Affiliations:** 1 Institute of Research in Immunology and Cancer (IRIC), Université de Montréal, Montréal, Québec, Canada; 2 Department of Pathology and Cell Biology, Université de Montréal, Montréal, Québec, Canada; 3 Department of Molecular Biology and Biochemistry, Rutgers University, Piscataway, New Jersey, United States of America; 4 Department of Physics and Astronomy, University of Denver, Denver, Colorado, United States of America; Baylor University, United States of America

## Abstract

In all organisms, cell polarity is fundamental for most aspects of cell physiology. In many species and cell types, it is controlled by the evolutionarily conserved PAR-3, PAR-6 and aPKC proteins, which are asymmetrically localized at the cell cortex where they define specific domains. While PAR proteins define the antero-posterior axis of the early *C. elegans* embryo, the mechanism controlling their asymmetric localization is not fully understood. Here we studied the role of endocytic regulators in embryonic polarization and asymmetric division. We found that depleting the early endosome regulator RAB-5 results in polarity-related phenotypes in the early embryo. Using Total Internal Reflection Fluorescence (TIRF) microscopy, we observed that PAR-6 is localized at the cell cortex in highly dynamic puncta and depleting RAB-5 decreased PAR-6 cortical dynamics during the polarity maintenance phase. Depletion of RAB-5 also increased PAR-6 association with clathrin heavy chain (CHC-1) and this increase depended on the presence of the GTPase dynamin, an upstream regulator of endocytosis. Interestingly, further analysis indicated that loss of RAB-5 leads to a disorganization of the actin cytoskeleton and that this occurs independently of dynamin activity. Our results indicate that RAB-5 promotes *C. elegans* embryonic polarity in both dynamin-dependent and -independent manners, by controlling PAR-6 localization and cortical dynamics through the regulation of its association with the cell cortex and the organization of the actin cytoskeleton.

## Introduction

Formation of polarized domains within a cell, through the asymmetric partition of proteins, lipids and RNAs, is required for a number of processes such as asymmetric cell division, cell migration, morphogenesis and maintenance of tissue architecture (reviewed in [1], [2]). PAR proteins constitute an evolutionarily conserved molecular machinery that is essential for the formation and maintenance of such polarized domains [3]. Most PAR proteins display a cortical and polarized localization that is crucial for their function. The three proteins PAR-3, PAR-6 and aPKC/PKC-3 (collectively referred to as anterior PAR proteins) can form a complex that localizes asymmetrically at the cortex of many different polarized cells. In the early *C. elegans* embryo, the anterior localization and the size of the domain occupied by the anterior PAR proteins are governed by actomyosin-dependent cortical flows, which initiate soon after fertilization and relocalize cortical components from the posterior to the anterior pole of the embryo [4]. Exclusion of the anterior PAR proteins from the posterior cortex by these cortical flows allows the recruitment of two other PAR proteins, PAR-1 and PAR-2, to the posterior cortex. Once localized, anterior and posterior PAR proteins mutually exclude one-another during the polarity maintenance phase, until the asymmetric division of the zygote. Although some anterior PAR proteins have been shown to interact with the membrane or with the underlying actin cytoskeleton in other cell types [2], the mechanisms that promote their asymmetric enrichment and dynamically regulate their cortical anchoring in *C. elegans* are unclear. In addition, the genes responsible to determine the proper location and size of the domain occupied by these proteins are largely unknown.

Over the past years, several studies have shed light on the role of endocytic components in the control of cell polarity [5], [6]. For instance, Rab5 is required for PAR protein cortical localization and for epithelial polarity in *Drosophila* [7]. While a recent study has demonstrated that the GTPase dynamin, an upstream regulator of endocytosis, affects the polarization of the *C. elegans* embryo [8], the contribution of other endocytic regulators in this process is unclear. Here, we studied the function of endocytic regulators in polarization of the *C. elegans* embryo. We find that depleting RAB-5, a small GTPase that functions as the main regulator of early endosome formation, perturbs the organization and function of PAR-6 in *C. elegans* during the maintenance phase of polarity. Interestingly, while depletion of RAB-5 recapitulated some of the phenotypes observed in embryos depleted for dynamin, it also resulted in unique spindle positioning and cortical organization defects. Our results support a model in which RAB-5 controls *C. elegans* early embryonic polarity in both dynamin-dependent and -independent manners, by regulating the cortical localization of PAR-6 and the organization of the actin cytoskeleton.

## Results

### Depletion of RAB-5 and other endocytic regulators results in early embryonic polarity phenotypes

**Figure 1:**
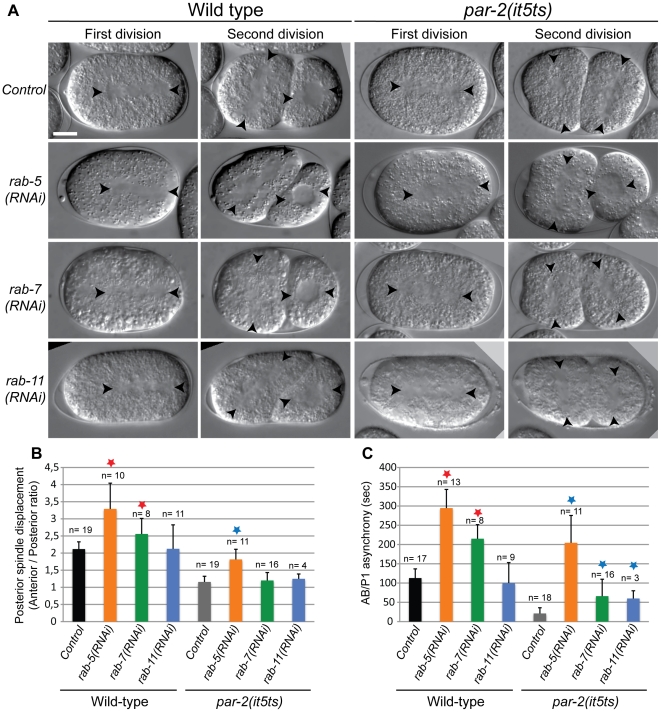
Depletion of RAB-5 and other endocytic regulators results in polarity phenotypes in early *C. elegans* embryos. (**A**) Midplane DIC images from time-lapse acquisitions of developing wild-type or *par-2(it5ts)* embryos undergoing first or second division with the indicated RNAi treatment. All of the embryos depicted successfully completed their first and second cytokineses. In each frame, anterior is to the left and arrowheads indicate centrosome position. Scale bar, 10 µm. (**B**) Quantitation of posterior spindle displacement in zygotes of each background, as determined by measuring the ratio between the position of anterior vs posterior centrosome. (**C**) Quantitation of the asynchrony between AB and P_1_ division at the 2-cell stage, as determined by measuring the time difference between cytokinesis onset in each cell. Stars indicate statistical significance (p<0.05, Student's t-test) when compared to *control(RNAi)* in wild type (red stars) or *par-2(it5ts)* backgrounds (blue stars). Depleting RAB-5 modulates all polarity-related phenotypes that were quantitated. n, number of embryos analyzed. Error bars represent standard deviation.

We hypothesized that endocytic regulators could contribute to early *C. elegans* embryonic polarization by regulating the cortical localization and/or function of anterior PAR proteins. To characterize this involvement, we focused our analysis on three small GTPases, known to be essential regulators of endocytic trafficking, RAB-5, RAB-7 and RAB-11, which respectively function as master regulators of early, late and recycling endosome function [9]. We first depleted each of these three genes by RNAi and monitored the first two divisions of *C. elegans* embryos by time-lapse differential interference contrast (DIC) microscopy, quantitating two polarity related phenotypes: positioning of the first mitotic spindle, which is typically displaced toward the posterior pole of the cell, and division asynchrony of the AB and P_1_ blastomeres at the two-cell stage, as AB typically divides 120 seconds before P_1_ in wild-type embryos. We found that depletion of RAB-5 or RAB-7 in a wild-type background affected both phenotypes and resulted in the first mitotic spindle forming more toward the posterior pole and in an increase in the asynchrony between AB and P_1_ division as compared to wild-type embryos (Figure 1). As previously reported [10], depletion of RAB-11 also affected both phenotypes, although the penetrance was more variable, with some embryos having their first spindle overly shifted to the posterior, as for RAB-5-depleted embryos, and other embryos showing defects consistent with a loss of polarity. These results indicate that depletion of RAB-5, RAB-7 and RAB-11 regulate polarity-dependent processes in the early *C. elegans* embryo.

Previous studies showed that regulators of *C. elegans* polarity can be revealed by functionally assessing whether their depletion can modify the embryonic phenotype of *par-2(it5ts)* mutants [11], [12], [13], [14]. For instance, depleting genes that positively regulate the amount of PAR-6 protein can result in suppression *par-2* mutant phenotypes, while negative regulators enhance the defects of *par-2* mutants. We therefore scored spindle positioning and cell division timing after depletion of each of the three endocytic regulators in *par-2(it5ts)* mutant embryos, in which the first mitotic spindle is central and, at the two-cell stage, the blastomeres divide synchronously (Figure 1). We found that depletion of RAB-5 efficiently suppressed these polarity defects in *par-2(it5ts)* mutant embryos grown at restrictive temperature (Figure 1), making it one of the strongest suppressors of *par-2* defects identified to date [14]. Depletion of either RAB-7 or RAB-11 in *par-2(it5ts)* mutants modified the cell division synchrony phenotype *of par-2(it5ts*) mutants but had no influence on the spindle positioning defect (Figure 1). Together, these results indicate that while several endocytic regulators contribute to polarization of the *C. elegans* embryo, RAB-5 could play a functionally important role in this process and we therefore focused our analysis on the role of this protein in embryonic polarization.

### RAB-5 controls PAR-6 cortical localization independently of actomyosin contractility

**Figure 2:**
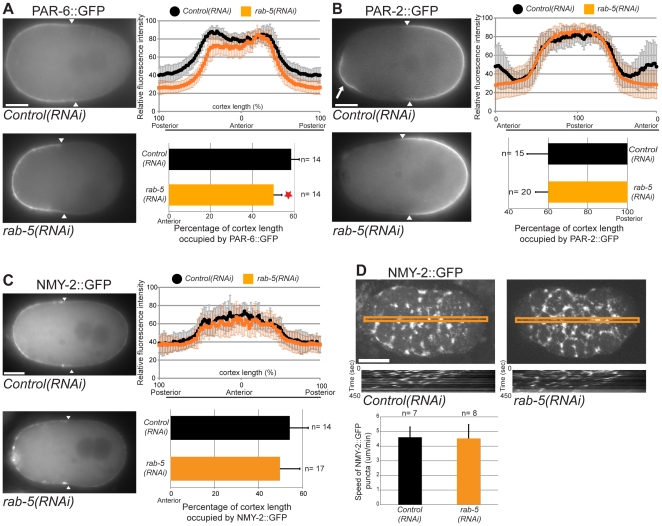
RAB-5 controls PAR-6 cortical localization independently of actomyosin contractility. (**A**) Midplane images of PAR-6::GFP in *control(RNAi)* and *rab-5(RNAi)* embryos at pronuclear meeting, i.e., at the end of the establishment phase of polarity. Quantitation of fluorescence intensity along the circumference of the cortex reveals that the size of PAR-6::GFP cortical domain is more anterior in *rab-5(RNAi)* embryos compared to *control(RNAi)* (red star, p = 6.11×10^−06^, Student's t-test). (**B**) Midplane images of GFP::PAR-2 in *control(RNAi)* and *rab-5(RNAi)* embryos at pronuclear meeting. Quantitation of fluorescence intensity along the cortex shows that the size of GFP::PAR-2 cortical domain is similar in *control(RNAi)* and *rab-5(RNAi)* embryos (p = 0.98, Student's t-test). Quantitation excluded the anterior crescent of GFP::PAR-2 visible in wild-type embryos (arrow) but absent from *rab-5(RNAi*) embryos. (**C**) Images of NMY-2::GFP at the mid-plane of *control(RNAi)* and *rab-5(RNAi)* embryos at pronuclear meeting. Quantitation of fluorescence intensity along the cortex shows that the size of NMY-2::GFP cortical domain is similar in *control(RNAi)* and *rab-5(RNAi)* embryos (p = 0.17, Student's t-test). (**D**) Images of NMY-2::GFP at the cortex of *control(RNAi)* and *rab-5(RNAi)* embryos during the phase of establishment of polarity. Kymographs were generated along the antero-posterior axis (orange lines). The average velocity of NMY-2 puncta is similar in *control(RNAi)* and *rab-5(RNAi)* embryos (p = 0.86, Student's t-test). In each frame anterior is to the left and white arrowheads indicate the boundary of cortical fluorescence. n, number of embryos analyzed. Error bars represent standard deviation. Scale bars, 10 µm.

As polarization of the early embryo depends on the asymmetric localization of PAR proteins, we tested whether depletion of RAB-5 by RNAi (Figure S1) perturbed the localization of anterior and posterior PAR protein cortical domains. We measured the distribution of PAR-6::GFP or GFP::PAR-2 at the time of pronuclear meeting, i.e., at the onset of the polarity maintenance phase, by quantitating the fluorescence intensity of each GFP tagged protein along the cortex (Figure 2). We found that PAR-6::GFP occupies 59% of the anterior cortex of wild-type embryos, as previously described [13], [15]. Interestingly, PAR-6::GFP localized to a smaller domain in embryos depleted of RAB-5 and covered 50% of the anterior cortex (Figure 2A), a phenotype that was not observed in embryos depleted for RAB-7 or RAB-11 (Figure S2). While anterior enrichment of endogenous PAR-6 was also observed by immunofluorescence on fixed *rab-5(RNAi)* specimen, no difference was observed for the localization of endogenous PKC-3 between *control(RNAi)* and *rab-5(RNAi)* embryos (Figure S3), and, the length of the PAR-2::GFP domain was also similar in *control(RNAi)* and *rab-5(RNAi)* embryos (Figure 2B). These results suggest that RAB-5 affects the localization of PAR-6 independently of other PAR proteins (see discussion). In addition to its posterior localization, PAR-2 is transiently present at the anterior cortex near the site of meiotic cytokinesis in 42% of wild-type embryos (n = 12), as previously reported [16], [17]. This anterior PAR-2 cap was observed in only 11% of *rab-5(RNAi)* embryos (n = 18). These results indicate that RAB-5 acts as a positive regulator of PAR-6 to regulate its activity and localization along the antero-posterior axis.

Localization of PAR-6 to the anterior cortex relies on cortical flows driven from the posterior cortex to the anterior cortex by non-muscle myosin II (NMY-2) activity [4]. Therefore, it is possible that depletion of RAB-5 results in an over-anteriorized PAR-6 domain by affecting cortical flows. To test this possibility, we measured the distribution of NMY-2::GFP along the antero-posterior axis of the embryonic cortex at pronuclear meeting. In *control(RNAi)* embryos, NMY-2 is enriched at the anterior of the embryo at the end of the polarization phase [4] and we found that this phenotype was not affected by depletion of RAB-5 (Figure 2C). We also quantitated the velocity of cortical NMY-2::GFP movement toward the anterior pole in time-lapse acquisitions and found no difference between *control(RNAi)* and *rab-5(RNAi)* embryos, in both of which myosin moved at an average speed of 4.5 µm/min (Figure 2D). Together, these results indicate that RAB-5 controls the anterior localization of PAR-6 independently of cortical NMY-2 flows during the polarity establishment phase.

### RAB-5 regulates PAR-6 residence time at the cortex

**Figure 3:**
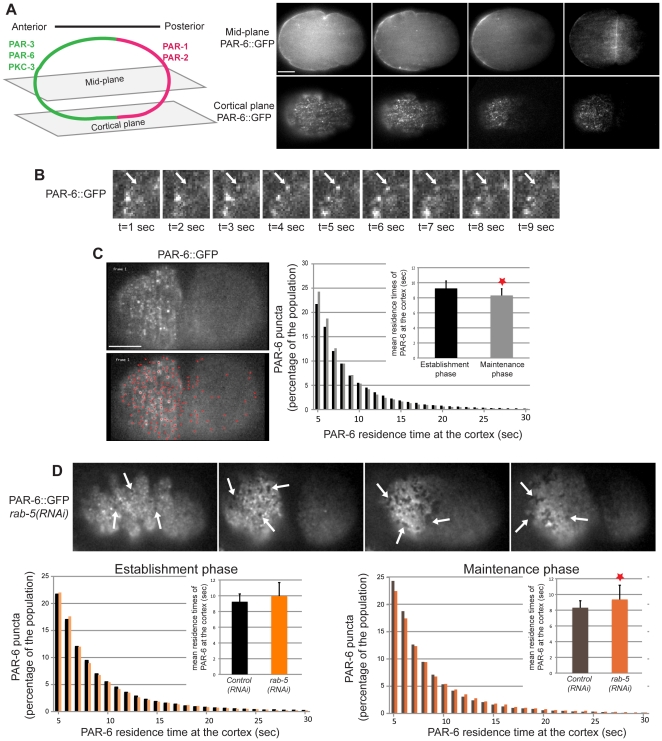
RAB-5 controls PAR-6 residence time at the cortex. (**A**) Time lapse images of PAR-6::GFP in the middle plane and cortical plane of *control(RNAi)* embryos. Imaging at the cortical plane was done by TIRF microscopy. A schematic representation of PAR protein localization in each plane is depicted on the left. (**B**) Magnified images of PAR-6::GFP at the cortex of *control(RNAi)* embryos obtained by TIRF microscopy. The white arrow points to a PAR-6-positive puncta appearing and disappearing from the focal plane during this 9-second excerpt. (**C**) TIRF images of cortical PAR-6::GFP in a *control(RNAi)* embryo before (upper image) and after (lower image) processing by particle tracking software. Each red dot on the bottom image is recognized as a PAR-6-positive structure. Quantitative automated analysis of PAR-6::GFP puncta shows that they have short cortical residence time during both the establishment and maintenance phases of polarity. The mean cortical residence time is significantly longer during the establishment phase than during the maintenance phase (red star, p = 0.003, Student's t-test). Error bars represent standard deviation. (**D**) TIRF images of cortical PAR-6::GFP in *rab-5(RNAi)* embryos. Quantitative automated analysis of PAR-6::GFP puncta shows that depletion of RAB-5 results in a significant increase in their mean cortical residence time during the maintenance phase (red star, p = 0.026, Student's t-test), but not during the establishment phase of polarity. Error bars represent standard deviation. White arrows point to cortical regions where PAR-6::GFP is excluded. In all panels anterior is to the left. Scale bars, 10 µm.

In *C. elegans* and other systems, RAB-5 was shown to be a critical regulator of endocytosis by controlling the formation and function of early endosomes. We therefore hypothesized that RAB-5 promotes PAR-6 localization through its role in the regulation of early endosome function. To test this, we devised a new method, based on total internal reflection fluorescence (TIRF) microscopy, to monitor the trafficking of PAR-6::GFP at the cell cortex. TIRF microscopy allows the illumination and detection of fluorophores that are present within 200 nm of the coverslip, revealing events that occur specifically at the cell cortex with high resolution. While PAR-6 distribution appears uniform and continuous when observed in mid-plane imaging sections [17], it was previously reported to localize to puncta when observed in cortical imaging planes [4], [15] (Figure 3A). As shown in Figure 3A and 3B, TIRF imaging revealed that PAR-6::GFP is visible in early embryos in individual puncta of various sizes and intensities that appear and disappear from the focal plane in a highly dynamic manner (Figure 3B and Movies S1, S2). We quantitated PAR-6 cortical dynamics using an automated computational approach based on tracking individual PAR-6::GFP puncta (Figure 3C and Movie S3). This approach allowed us to simultaneously track hundreds of puncta per embryo. We found that PAR-6::GFP puncta displayed on average very short residence times at the cortex. The cortical residence time of PAR-6::GFP was significantly longer during the establishment phase of polarity (9.2±1.0 sec) than during the maintenance phase (8.3±0.9 sec; p = 0.003), consistent with the hypothesis that these two phases are governed by distinct mechanisms (Figure 3C). Interestingly, depleting RAB-5 perturbed the dynamics of PAR-6::GFP puncta during the maintenance phase of polarity and resulted in an increase in their residence times at the cortex (and perhaps also their overall size) when compared to *control(RNAi)* embryos (9.4±1.8 sec; p = 0.025) (Figure 3D and Movies S4, S5). RAB-5 depletion had no statistically significant effect on cortical PAR-6::GFP dynamics during the establishment phase of polarity (10.0±1.7 sec; p = 0.112), suggesting that RAB-5 activity contributes to polarity mainly during the maintenance phase. Therefore, we conclude that PAR-6 is a highly dynamic cortical protein whose cortical residence time is controlled by RAB-5 during embryonic polarization.

### PAR-6 partially co-localizes with endocytic markers at the cortex and in the cytoplasm

**Figure 4:**
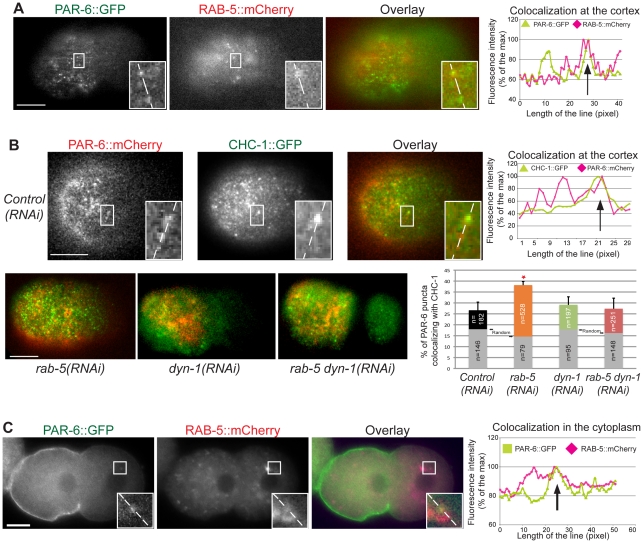
PAR-6 co-localizes with endocytic markers at the cortex and in the cytoplasm. (**A**) Images of PAR-6::GFP and mCherry::RAB-5 at the cortex of *control(RNAi)* embryos during the polarity maintenance phase. The box is magnified three-fold in inset. Fluorescence intensity was measured along the line in each inset and represented for PAR-6 (green) or RAB-5 (red). The arrow points to a region with peaks of fluorescence intensity for each channel, and thus co-localization for both markers. (**B**) Images of PAR-6::mCherry and GFP::CHC-1 at the cortex of *control(RNAi)*, *rab-5(RNAi)*, *dyn-1(RNAi)* and *rab-5(RNAi); dyn-1(RNAi)* embryos during the polarity maintenance phase. Graphical representations are as in panel A, except that PAR-6 is in red and CHC-1 is in green. The bar graph shows that co-localization of PAR-6::mCherry with GFP::CHC-1 is significantly increased in *rab-5(RNAi)* embryos compared to *control(RNAi)* (red star, p = 0.022, Student's t-test) but not in *dyn-1(RNAi)* (p = 0.7) or in *rab-5(RNAi); dyn-1(RNAi)* (p = 0.89). The gray bars represent random co-localization control. n, number of puncta analyzed. Bars represent standard error of the mean. (**C**) Midplane images of PAR-6::GFP and mCherry::RAB-5 in the cytoplasm of *control(RNAi)* embryos during the polarity maintenance phase. Graphic representations are as in panel A. In all panels, anterior is to the left. Scale bars, 10 µm.

Among the proteins present at the cortex, components of the early endocytic pathway have been shown to be extremely dynamic with high variance in residence times. In mammals for instance, such components are typically visible by TIRF microscopy for periods ranging from 5 to 100 seconds [18]. Furthermore, in *C. elegans*, some endocytic regulators are known to be enriched at the anterior of the embryo where anterior PAR proteins localize [19]. Therefore the high, RAB-5-dependent cortical dynamics of PAR-6 could reflect an important association of the PAR proteins at the cortex with endocytic organelles. To address this possibility, we generated double transgenic strains co-expressing fluorescently-tagged PAR-6 (GFP or mCherry) with RAB-5 fused to mCherry (mCherry::RAB-5) or clathrin heavy chain fused to GFP (GFP::CHC-1), and used TIRF microscopy to analyze their co-localization at the cortex during the polarity maintenance phase. One caveat is that our analyses may be based on protein overexpression, although this is typically mild in the *C. elegans* germline and embryo. Quantitation of fluorescence intensities revealed that 18.8±1.4% of PAR-6::GFP puncta co-localized with mCherry::RAB-5 at the cortex (Figure 4A; 182 puncta, 4 embryos). This was significantly higher than the control (6.3±1.4%; 64 puncta, 4 embryos; p = 0.0002) in which random co-localization was determined using the same analysis performed with one of the two images flipped horizontally. By contrast, we observed that 26.6±3.7% of PAR-6::mCherry puncta co-localized with GFP::CHC-1 positive structures (Figure 4B; 528 puncta, 5 embryos), a value that is higher but not statistically significantly different than the random co-localization control (17.9±2.6%; 146 puncta, 5 embryos, p = 0.09). Interestingly, depletion of RAB-5 resulted in a significant increase in PAR-6::mCherry and GFP::CHC-1 co-localization compared to *control(RNAi)* (Figure 4B; 38.1±1.7%, 651 puncta, 5 embryos; p = 0.02). This was significantly higher than random co-localization control (14.6±3.9%; 79 puncta, 4 embryos, p = 0.0006), indicating that the increase in co-localization is specific for RAB-5 depletion. These results indicate that RAB-5 regulates the association of PAR-6 with early endocytic regulators at the cortex.

While PAR-6::GFP mainly localizes to the cortex in early embryos, we observed that low amounts are also visible as cytoplasmic puncta during the polarization phase. Interestingly, we found that 37.9% of PAR-6::GFP cytoplasmic puncta co-localized with mCherry::RAB-5 (Figure 4C; 49 puncta, 11 embryos), a value significantly higher than random co-localization control (9.3±4.7%; 49 puncta, 3 embryos). PAR-6 also partially co-localized in the cytoplasm with EEA-1, another marker of early endosomes (data not shown). Furthermore, immunofluorescence experiments on fixed specimens revealed that some of these PAR-6::GFP cytoplasmic structures can also contain PAR-3 and PKC-3, suggesting that all anterior PAR proteins can be found in endosomal compartments (Figure S4). We did not observe any co-localization of PAR-6::GFP with either mCherry::RAB-7 or mCherry::RAB-11, indicating specificity of PAR-6 association with the early endosome compartment (Figure S4). These results indicate that PAR-6 and possibly other anterior PAR proteins associate with early endosomes during embryonic polarization and that such association is likely to be important for the regulation of polarity.

### RAB-5 controls actin and PAR-6 organization at the cortex

**Figure 5:**
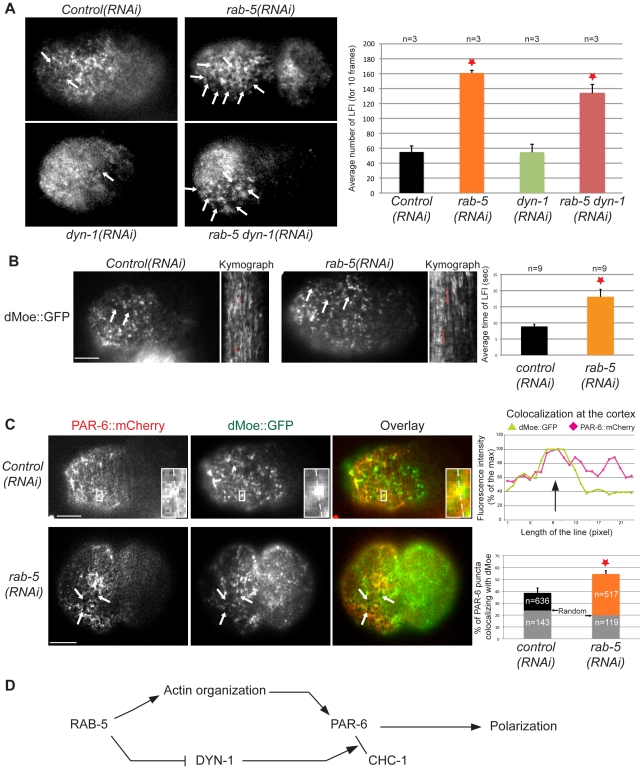
RAB-5 controls actin and PAR-6 organization at the cortex. (**A**) Images of dMoe::GFP at the cortex of *control(RNAi)*, *rab-5(RNAi)*, *dyn-1(RNAi)* and *rab-5(RNAi); dyn-1 (RNAi)* embryos during the phase of maintenance of polarity. Regions of low fluorescence intensity (LFI) are visible in all backgrounds (white arrows), but quantitation reveals that they are more frequent in *rab-5(RNAi)* and *rab-5(RNAi); dyn-1(RNAi)* embryos (red star, compared to *control(RNAi)*: p = 0.00027 and p = 0.0054 respectively, Student's t-test). n, number of embryos analyzed. Error bars represent standard error of the mean. (**B**) Images of dMoe::GFP at the cortex of *control(RNAi)* and *rab-5(RNAi)* embryos during the phase of maintenance of polarity. Timed quantitation by kymograph analysis shows that the LFI regions are visible for a longer time in *rab-5(RNAi)* embryos (red star, p = 0.037, Student's t-test). n, number of embryos analyzed. Error bars represent standard error of the mean. (**C**) Images of PAR-6::mCherry and dMoe::GFP at the cortex of *control(RNAi)* and *rab-5(RNAi)* embryos during the phase of maintenance of polarity. The box is magnified four-fold in inset. Fluorescence intensity was measured along the line in each inset and represented for PAR-6 (red) or dMoe (green). The black arrow in the graph points to a region with peaks of fluorescence intensity for each channel, and thus co-localization for both markers. The bar graph shows that co-localization of PAR-6::mCherry with dMoe::GFP is significantly increased in *rab-5(RNAi)* embryos compared to *control(RNAi)* (red star, p = 0.013, Student's t-test). The gray bars represent random control co-localization. n, number of puncta analyzed. Bars represent standard error of the mean. White arrows point to regions of LFI that are visible with both markers and can be superimposed. (**D**) Model depicting the regulation of PAR-6 function by RAB-5 (See discussion for details).

In addition to its role in early endocytosis and in the function of early endosomes, RAB-5 has been shown to control actin remodeling in mammalian cells [20], [21]. Depletion of *C. elegans* RAB-5 was also reported to mildly affect the actomyosin cytoskeleton in addition to severely perturbing the morphology of the endoplasmic reticulum [22]. As the actin cytoskeleton typically mediates the cortical organization of PAR proteins, we hypothesized that RAB-5 may control *C. elegans* embryonic polarity through its role in actin remodeling. Indeed, we observed that PAR-6::GFP cortical organization is perturbed in *rab-5(RNAi)* embryos, in which it appears to be excluded from sub-regions of the anterior cortex during the maintenance phase of polarity (Figure 3D, arrows). We therefore analyzed the effect of RAB-5 depletion on actin organization. TIRF imaging of embryos expressing the actin-binding domain of dMoesin fused to GFP (dMoe::GFP; [23]) revealed that RAB-5 depletion results in disorganization of the actin cytoskeleton. Specifically, dMoe::GFP qualitatively appeared less filamentous and more punctate than in *control(RNAi)* embryos. After depletion of RAB-5, we also observed the appearance of several regions of lower dMoe::GFP fluorescence intensity (Figure 5A). These regions were observed more frequently in *rab-5(RNAi)* embryos (161±4 regions per 10 frames) than in *control(RNAi)* (55±8 regions per 10 frames; p = 0.0003). Kymograph analysis revealed that these regions are dynamic in both conditions but that their dynamics are slower in *rab-5(RNAi)* embryos compared to *control(RNAi)* (Figure 5B and Movies S6, S7). Such disorganization was mainly observed during the maintenance phase of polarity, after completion of the contractility-based polarity establishment phase. A similar cortical disorganization was also observed at the end of the polarity establishment phase in RAB-5-depleted embryos expressing NMY-2::GFP (Figure 2C). This demonstrates that *rab-5* depletion leads to a disorganization of cortical PAR-6 and, more generally, of the actin cytoskeleton.

To determine the links between the organization of PAR-6 and the actin cytoskeleton, we generated a strain co-expressing PAR-6::mCherry and dMoe::GFP and monitored the localization of both cortical markers during the polarity maintenance phase using TIRF microscopy. We found that 38.7%±4.1 of the PAR-6::mCherry puncta co-localized with dMoe::GFP (Figure 5C; 636 puncta, 6 embryos) and this was significantly higher than the random control (23.8%±2.3; 143 puncta, 6 embryos; p = 0.01). Co-localization of PAR-6 and dMoe was significantly increased in *rab-5(RNAi)* embryos when compared to *control(RNAi)* (Figure 5C; 54.6±6.2%, 517 puncta, 5 embryos; p = 0.01), whereas no change was found when measuring co-localization in the random control samples (19.9%±2.8; 119 puncta, 5 embryos; p = 0.29). This indicates that depletion of RAB-5 increases the co-localization between PAR-6 and F-actin. Furthermore, we observed that the regions of low dMoe::GFP fluorescence intensity were also largely devoid of PAR-6::mCherry fluorescence signal (Figure 5C, arrows). This indicates that RAB-5 could regulate PAR-6 organization and dynamics by controlling its association with F-actin and the general organization of the actin cytoskeleton.

### RAB-5 has DYN-1-dependent and –independent functions in the early embryo

Our results support a role for RAB-5 in the regulation of PAR-6 cortical dynamics during *C. elegans* embryonic polarization. DYN-1, the *C. elegans* ortholog of dynamin, has been reported to exist in a complex with RAB-5 [24], and was previously implicated in regulation of embryonic polarity [8], suggesting a broader involvement of endocytic trafficking in this process. This is further supported by the report that turnover of cortical PAR-6 is slower in *dyn-1(RNAi)* embryos [8], which is compatible with our finding that PAR-6 cortical residence time is controlled by RAB-5 during embryonic polarization. However, the spindle positioning defect that we observed in *rab-5(RNAi)* embryos is distinct from that following depletion of DYN-1, and a recent report demonstrated that RAB-5 controls spindle positioning independently of DYN-1 [25]. We therefore determined whether other RAB-5-dependent phenotypes monitored in our analysis depend on DYN-1 function. We first tested whether the increase in PAR-6::mCherry and GFP::CHC-1 co-localization observed after depletion of RAB-5 is also observed after depleting DYN-1. We found that these two proteins co-localized to comparable levels in DYN-1-depleted embryos compared to *control(RNAi)* (Figure 4B; 29.1±4.8%, 197 puncta, 6 embryos for *dyn-1(RNAi)* compared to 26.6±3.7%, 528 puncta, 5 embryos for *control(RNAi)*; p = 0.7), indicating that depleting DYN-1 alone does not modify their co-localization. However, the *rab-5(RNAi)*-dependent increase in PAR-6::mCherry and GFP::CHC-1 cortical co-localization was lost in embryos co-depleted for both RAB-5 and DYN-1 (Figure 4B; 27.4±3.6%, 251 puncta, 5 embryos; p = 0.89 when compared to *control(RNAi)* and p = 0,027 when compared to *rab-5(RNAi)*). This indicates that the increase in PAR-6::mCherry and GFP::CHC-1 co-localization observed after RAB-5 depletion requires the function of DYN-1. It further demonstrates DYN-1 levels were duly depleted in *rab-5(RNAi); dyn-1(RNAi)* embryos.

We then determined whether depleting DYN-1 affected the general organization of the actin cytoskeleton, as is the case for RAB-5 depletion. We found that upon DYN-1 depletion, the number of regions with low dMoe::GFP fluorescence intensity was comparable to *control(RNAi)* embryos (55±11 regions; p = 0.98) and significantly lower than in *rab-5(RNAi)* embryos (p = 0.008; Figure 5A). Interestingly, these regions of dMoe::GFP fluorescence intensity were also observed in abnormally high numbers (134±12 regions) in embryos co-depleted for both DYN-1 and RAB-5, indicating that RAB-5 regulates the organization of the actin cytoskeleton independently of DYN-1. While it remains formally possible that, under our RNAi conditions, DYN-1 is sufficiently depleted to reveal its role in regulating the association between PAR-6 and CHC-1 but that further depletion would be required to observe effects on the actin cytoskeleton, we note that the polarity defects associated with DYN-1-depletion are observed under our RNAi conditions and therefore we consider this as unlikely. Together, these results support a model in which RAB-5 regulates the organization of the actin cytoskeleton independently of DYN-1 function.

## Discussion

Our findings reveal that RAB-5 plays a significant role in polarization of the early *C. elegans* embryo. We showed that depleting the endocytic regulator RAB-5 perturbs the size of PAR-6 domain during the polarity establishment phase, as well as PAR-6 dynamics and cortical organization during the phase of maintenance of polarity. Depletion of RAB-5 suppressed the polarity phenotypes of *par-2* mutants, indicating that its activity becomes critical when polarity is compromised and that its role in this process is biologically relevant. Furthermore, RAB-5 depletion disrupted the organization of cortical actin and altered the co-localization of PAR-6 with components such as clathrin heavy chain at the embryonic cortex. Based on these results, we propose that RAB-5 controls PAR-6 localization and cortical dynamics by regulating the organization of the actin cytoskeleton and the association of PAR-6 (and perhaps other anterior PAR proteins) with cortical components. Depleting RAB-5 did not perturb cortical NMY-2 flows at the posterior cortex during the establishment phase of polarity. On the other hand, it had a significant effect on the duration of PAR-6 residence time at the cortex during the polarity maintenance phase. Together, these results suggest that RAB-5 exerts its activity mainly during polarity maintenance. However, we note that PAR-6 localization was affected during the polarity establishment phase in *rab-5(RNAi)* embryos, indicating that RAB-5 could also act during this time. As anterior PAR proteins are themselves positive regulators of endocytosis [26], our results reflect the possible existence of a feedback mechanism between RAB-5 and PAR proteins to regulate polarity and actin organization.

Previous work showed that depletion of dynamin results in a small spindle-positioning defect in the 1-cell embryo and in a decrease in PAR-6 dynamics at the cortex [8]. Together with our quantitative analysis of PAR-6 association with early endosomal markers, this supports the notion that part of RAB-5's role in cell polarity occurs through its function in endocytosis and early endosome formation. However, several lines of evidence suggest that RAB-5 has a broader role in the regulation of cell polarity, as depleting dynamin did not recapitulate all of the defects observed following RAB-5 depletion. For instance, the posterior over-displacement phenotype of the spindle that we observed in RAB-5-depleted embryos is opposite from the anterior positioning defect reported for dynamin-depleted embryos [8], [25]. Furthermore, we found that the defects in PAR-6 and actin cortical organization observed after RAB-5 depletion were absent from embryos depleted in dynamin but were still visible in embryos co-depleted for RAB-5 and DYN-1, indicating that RAB-5 controls cortical organization independently of DYN-1 function. We therefore propose a model (Figure 5D) in which RAB-5, along with dynamin, controls polarity through its function as an endocytic regulator by regulating the cortical trafficking of PAR-6, but has other, dynamin-independent functions in ensuring correct spindle positioning and in maintaining proper actin and PAR-6 cortical organization. While it is currently unclear how this additional, dynamin-independent function of RAB-5 in cell polarity is related to its role in endocytosis, it is compatible with the idea of early endosomes acting as hubs for intracellular signaling, as has been recently suggested [27].

Our results revealed that PAR-6 co-localizes robustly with clathrin heavy chain at the cortex of *rab-5(RNAi)* embryos. While we also observed co-localization between these two proteins in wild-type embryos, the value was not significantly different from random co-localization control measurements (p = 0.09). This suggests that the association of PAR-6 with the early endocytic machinery is transient and highly dynamic in wild-type embryos, and that depletion of RAB-5 increases the time of association in a dynamin-dependent manner. This is compatible with the role of RAB-5 in regulating early endosome formation and endocytic trafficking. Alternatively, it is possible that depleting RAB-5 confers novel properties to cortical components that would promote such association.

Our results revealed that RAB-5 controls the anterior localization of PAR-6 but has no effect on the localization of PAR-2. This is surprising given that anterior and posterior PAR proteins have been shown to localize to mutually exclusive cortical domains. Why does PAR-2 posterior localization remain unchanged despite the over-anteriorization of PAR-6? We found that cortical flows are normal in RAB-5-depleted embryos, suggesting that polarization occurs normally and that most defects are observed during the maintenance phase of polarity. This leads us to speculate that RAB-5 would not be required for the localization of PAR-2 during the polarization phase, but would be necessary for its trafficking dynamics during maintenance phase, such that its localization to cortices that are free of PAR-6 would be more difficult without RAB-5 function, and perhaps endocytosis function. The possibility that RAB-5 is involved in the trafficking of PAR-2 is further supported by our observation that the transient anterior PAR-2 crescent is lost in *rab-5(RNAi)* embryos. Such anterior accumulations of PAR-2 were previously reported to depend on the meiotic spindles and the mid-body remnants following meiotic cytokineses [28], and our results suggest that their presence could require dynamic endocytosis. Alternatively, we found that PKC-3 localization is normal in RAB-5-depleted embryos, and therefore it is possible that PAR-2 is prevented from spreading toward the anterior cortex by PKC-3 activity despite PAR-6 being displaced toward the anterior. Such scenario would indicate that PKC-3 can function without PAR-6 at the cortex of RAB-5 depleted embryos. Additional experiments will be required to further address these issues.

Finally, our results revealed that the cortical residence time of PAR-6 is short during both establishment and maintenance phases of embryonic polarity, and that depletion of RAB-5 results in a statistically significant increase in PAR-6 cortical residence time during the polarity maintenance phase. This suggests that a rapid turnover of cortical PAR-6 is required to accurately promote polarization. While it is currently unclear why a ∼13% difference would have such effect on polarity, we note PAR-6 maximal fluorescence recovery after photobleaching was reported to be lower in embryos depleted for DYN-1 [8], consistent with this process being co-regulated by both proteins.

## Materials and Methods

### Strains and alleles

All strains were maintained as described by Brenner [29]. They were grown at 15°C and assayed at 25°C after being grown at 25°C for at least 24 hours, unless otherwise stated. The wild-type strain was the N2 Bristol strain. The strains used in this study are presented Table S1. Double transgenic strains were generated by genetic crosses and each marker was followed either by phenotypic analysis or by PCR using specific primers. RNAi was performed on L4 animals using the feeding method as described [30], and worms were grown on RNAi plates for a minimum of 24 hours before collecting the embryos for phenotypic analysis. Except for RAB-5, whose depletion was directly determined by measuring fluorescence levels of a GFP fusion protein, the success of RNAi depletion for all proteins was based on assessing whether the resulting phenotype recapitulated published defects.

### Image acquisition

For all imaging methods, embryos were obtained by cutting open gravid hermaphrodites using two 25-gauge needles and mounted individually using a mouth pipette on a coverslip coated with 0.1% poly-l-lysine in 10 µl of egg buffer [31]. The coverslip was placed on a 2% agarose pad and the edges were sealed with petroleum jelly.

Detection of fluorescent proteins at the embryonic cortex was done using TIRF microscopy. Images were acquired by a Cascade II camera (Photometrics, Tucson, AZ, USA) mounted on a Nikon Eclipse Ti microscope (Nikon Canada Inc, Mississauga, ON, Canada). Samples were illuminated by 10 mW solid state lasers (488 nm and 561 nm, 10–30% power) and fluorescent light was collected by a Plan Apochromat 60×/1.49 NA objective. Images were acquired at 1 second intervals for single channel imaging and at 3 seconds intervals for dual channel imaging and acquisition was controlled by Elements software (Nikon).

Epifluorescence microscopy was used to measure the length of GFP::PAR-2, PAR-6::GFP or NMY-2::GFP cortical domains as well as the cytoplasmic co-localizations between PAR-6::GFP and either mCherry::RAB-5, mCherry::RAB-7 or mCherry::RAB-11. Images were acquired by a Zeiss HRM camera (Carl Zeiss Canada Ltd., Toronto, ON, Canada) mounted on a Zeiss Axio-Imager Z1 microscope. Samples were illuminated for 50–100 milliseconds by a mercury lamp and fluorescent light was collected by a Plan Apochromat 63×/1.4 NA objective. Images were acquired at 10 seconds intervals and the acquisition system was controlled by Axiovision software.

Swept Field Confocal (SFC) microscopy was used to image cortical NMY-2::GFP and measure the velocity of cortical flows. Images were acquired by a CoolSNAP HQ^2^ camera (Photometrics) mounted on a Nikon SFC microscope (Nikon and Prairie Technologies, Madison, WI, USA) using the 45 µm pinhole setting. Samples were illuminated by 10 mW solid state lasers (488 nm and 561 nm, 10–30% power) and fluorescent light was collected by a 100×/1.4 NA Plan-Apochromat objective was used to acquire 16 confocal sections (separated by 0.5 µm) of the upper cortex exposed for 80 milliseconds at 10 second intervals. The acquisition system was controlled by Elements software (Nikon).

Time-lapse Differential Interference Contrast (DIC) microscopy was done using Plan Apochromat 63×/1.4 NA or 100×/1.4 NA objectives mounted on a Zeiss Axioimager Z1 microscope, as described above. Images were acquired at 10 seconds intervals. Image analysis was performed using ImageJ software (http://rsbweb.nih.gov/ij/). Positioning of the first mitotic spindle and asynchrony between AB and P_1_ cells were quantitated as previously described [13].

### Image analysis

The co-localization between fluorescently-tagged PAR-6 and other fluorescent markers (CHC-1, RAB-5, RAB-7, RAB-11, dMoe) was determined using ImageJ software (NIH). A 10 pixel-thick line crossing a cortical or cytoplasmic PAR-6 punctum was drawn and mean fluorescence intensity profiles along this line were obtained and compared for both fluorescent channels. Proteins were deemed at co-localizing when a peak of PAR-6 fluorescence intensity was directly superimposed with that of the other fluorescent marker. Quantitation of random co-localization control was done similarly, except that the images were rotated along the horizontal axis for one of the two markers.

To quantitate the size of PAR-2, PAR-6, PKC-3 or NMY-2 cortical domains, ImageJ software was used to draw a 10 pixel-thick line on the whole perimeter of the embryos and plot the corresponding mean fluorescence intensity profiles. The mean fluorescence intensity values were interpolated as the percentage of total perimeter for each embryo. In all cases 0% = anterior pole and 100% = posterior pole. Intensities were represented as the percentage of maximum intensity along the perimeter. The length of domains were calculated as the length of embryo perimeter with intensity values superior to a certain threshold (30% of maximal intensity for PAR-6::GFP and NMY-2::GFP, 40% for PAR-6 and PKC-3 or 60% of maximal intensity for PAR-2::GFP). Depletion of RAB-5 by RNAi induced a discontinuity in the NMY-2::GFP domain, leading to the appearance of small cortical patches where fluorescence intensity was below the threshold within a region of high fluorescence intensity. These patches of low fluorescence intensity that were bordered by regions of higher fluorescence intensity were considered as superior to the threshold to properly determine domain length. In these embryos, fluorescence intensity below the threshold was therefore only considered significant when it was measured for a continuous region larger than 2 µm.

To quantitate the velocity of NMY-2 foci during polarization, we generated kymographs using ImageJ software by drawing a line from the anterior to the posterior pole of the embryos. Three kymographs were created for each embryo (one on each side and one in the center of the embryo) and the 12 most posterior foci present in these kymographs were used for quantitation. Velocity was calculated by reporting the distance traveled by these foci over time.

For fluorescence lifetime analysis, PAR-6 puncta were automatically detected and tracked as described [18]. The detection of puncta was set to be relatively sensitive, which results overall in fewer false negative and more frequent false positive detections. As a result, very short trajectories (≤3 frames), which are more likely to represent individual or linked false positive detections, were excluded from analysis. Lifetime distribution/histogram and mean lifetime were extracted for n = 20 embryos for each condition. While the lifetime distributions of individual embryos are not normal distributions, the distribution of mean lifetimes measured in different embryos is normally distributed due to the Central Limit Theorem. Thus, the significance of differences of mean lifetimes between individual conditions could be determined using Student's t-test.

For the quantitation of F-actin cortical organization, regions of low fluorescence intensity were manually counted on 10 frames (each separated by 10 seconds) obtained from TIRF movies. Three embryos of each genotype were analyzed. Quantitation of the lifetimes of these low fluorescence intensity regions was done using the same method used to determine NMY-2 velocity, but tracking the regions of low dMoe::GFP fluorescence intensity instead.

To measure spindle positioning, centrosome positions in DIC images were determined as described previously [13].

## Supporting Information

Figure S1 **RAB-5 can be efficiently depleted by RNAi in early embryos.** Midplane images of RAB-5::GFP in *control(RNAi)*, *rab-5(RNAi)* and *rab-5 dyn-1(RNAi)* embryos at pronuclear meeting, i.e., at the end of the phase of establishment of polarity. Quantitation of total cytoplasmic fluorescence intensity revealed a decrease in *rab-5(RNAi)* and in *rab-5 dyn-1(RNAi)* compared to control (p<0.001 in both cases, Student's t-test; red stars). In all panels, anterior is to the left. Scale bars, 10 µm.
(JPG)

Figure S2 **PAR-6 localization in** ***rab-7(RNAi)*** **and** ***rab-11(RNAi)*** **embryos.** Midplane images of PAR-6::GFP in *control(RNAi)*, *rab-7(RNAi)* and *rab-11(RNAi)* embryos at pronuclear meeting, i.e., at the end of the phase of establishment of polarity. Quantitation of fluorescence intensity along the cortex reveals that the size of PAR-6::GFP cortical domain is similar in control and *rab-7(RNAi)* (p = 0.77, Student's t-test) and enhanced in *rab-11(RNAi)* (p = 4.97×10^−05^, Student's t-test). In all panels, anterior is to the left. Scale bars, 10 µm.
(JPG)

Figure S3 **Localization of endogenous PAR-6 and PKC-3 in** ***rab-5(RNAi)*** **embryos.** Midplane images of *control(RNAi)* and *rab-5(RNAi)* embryos at pronuclear meeting, i.e., at the end of the polarity establishment phase, labelled with anti-PAR-6 or anti-PKC-3 antibodies. Quantitation of fluorescence intensity along the cortex revealed that the size of the PAR-6 cortical domain is reduced in *rab-5(RNAi)* embryos (p = 0.011, Student's t-test; red star) compared to *control(RNAi)* embryos whereas the size of the PKC-3 cortical domain is similar in *control(RNAi)* and *rab-5(RNAi)* embryos (p = 0.41, Student's t-test). In all panels, anterior is to the left. Scale bars, 10 µm.
(TIF)

Figure S4 **The PAR-3/PAR-6/PKC-3 complex localizes to cytoplasmic puncta.** (**A**) Midplane confocal images of fixed embryos expressing PAR-6::GFP and labelled with anti-PAR-3 and anti-PKC-3 antibodies during the phase of establishment of polarity. The white color indicates that all three markers co-localize. Inset shows magnification of the boxed region. In all frames, anterior is to the left. Scale bar, 10 µm. (**B**) Midplane images of PAR-6::GFP and mCherry::RAB-7 in the cytoplasm of control embryos during the phase of establishment of polarity. The box is magnified 2.5-fold in inset. Fluorescence intensity was measured along the lines in each inset and represented for PAR-6 (green) or RAB-7 (red). No significant co-localization was observed (3.2% of co-localization, n = 53 puncta) compared to random control co-localization (3.1%, n = 31). (**C**) Midplane images of PAR-6::GFP and mCherry::RAB-11 in the cytoplasm of control embryos during the phase of establishment of polarity. The box is magnified 2-fold in inset. Fluorescence intensity was measured along the lines in each inset and represented for PAR-6 (green) or RAB-11 (red). No co-localization was observed (3.8% of co-localization with RAB-11 (n = 47 puncta) compared to random control co-localization (0%, n = 21). In all panels, anterior is to the left. Scale bars, 10 µm.
(JPG)

Movie S1 **TIRF imaging of a wild-type embryo expressing PAR-6::GFP during the polarity establishment phase.** Images were captured at 1 second intervals and are played back at 10 images/second.
(MOV)

Movie S2 **TIRF imaging of a wild-type embryo expressing PAR-6::GFP during the polarity maintenance phase.** Images were captured at 1 second intervals and are played back at 10 images/second.
(MOV)

Movie S3 **TIRF imaging of a wild-type embryo expressing PAR-6::GFP in which the fluorescence tracking software has been applied.** Red spots correspond to fluorescence events. Images were captured at 1 second intervals and are played back at 10 images/second. Bar, 5 µm.
(MOV)

Movie S4 **TIRF imaging of a** ***rab-5(RNAi)*** **embryo expressing PAR-6::GFP during the polarity establishment phase.** Images were captured at 1 second intervals and are played back at 10 images/second.
(MOV)

Movie S5 **TIRF imaging of a** ***rab-5(RNAi)*** **embryo expressing PAR-6::GFP during the polarity maintenance phase.** Images were captured at 1 second intervals and are played back at 10 images/second.
(MOV)

Movie S6 **TIRF imaging of a wild-type embryo expressing dMoe::GFP during the polarity maintenance phase.** Images were captured at 1 second intervals and are played back at 10 images/second.
(MOV)

Movie S7 **TIRF imaging of a** ***rab-5(RNAi)*** **embryo expressing dMoe::GFP during the polarity maintenance phase.** Images were captured at 1 second intervals and are played back at 10 images/second.
(MOV)

Table S1 **Strains used in this study.**
(DOC)
