# Supplementary figures and images for: RAB-5 Controls the Cortical Organization and Dynamics of PAR Proteins to Maintain C. elegans Early Embryonic Polarity
Source: PLoS One. 2012 Apr 24;7(4):e35286. doi: 10.1371/journal.pone.0035286 (PMC3335856; doi:10.1371/journal.pone.0035286)

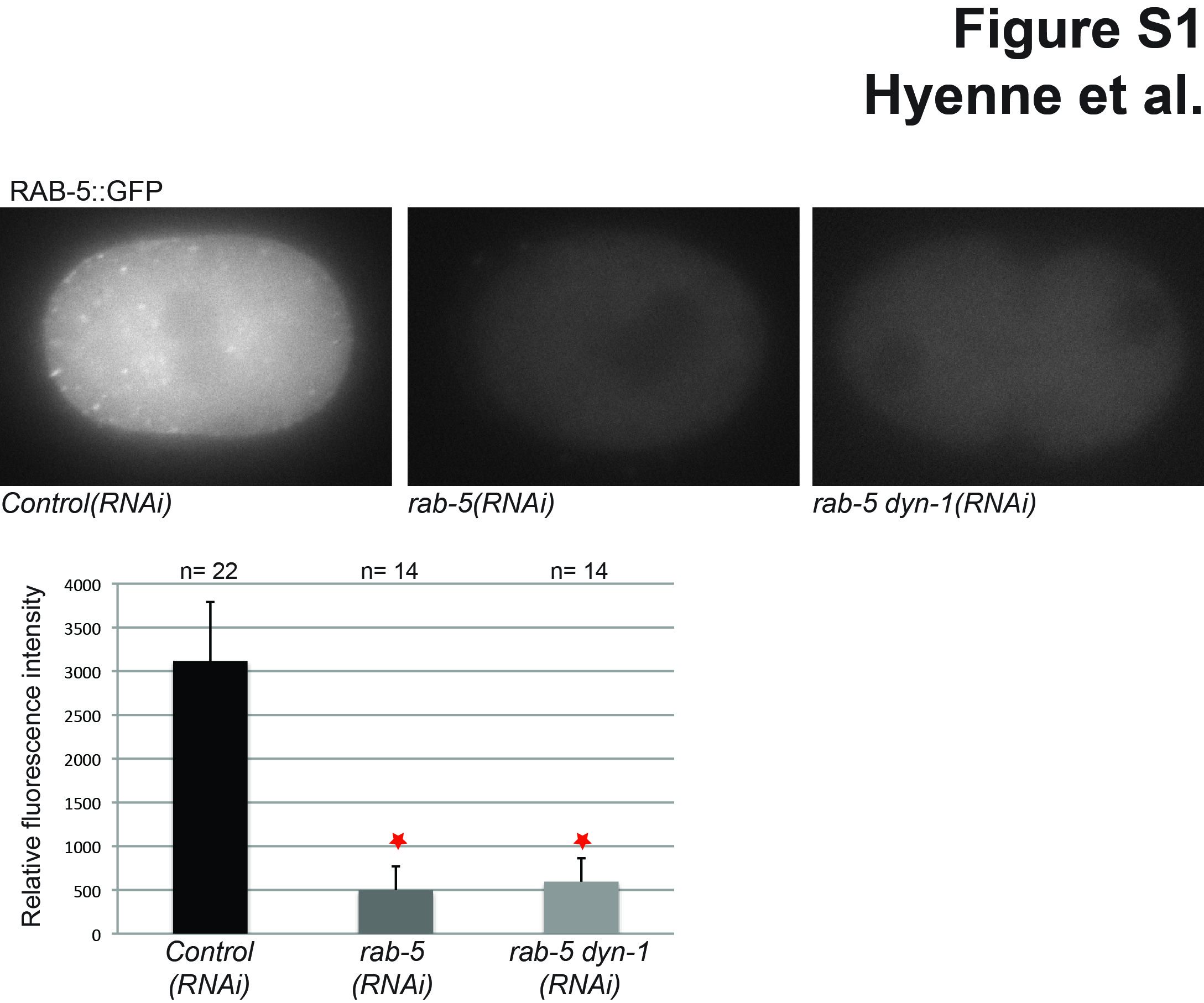

Supplement: Figure S1 — RAB-5 can be efficiently depleted by RNAi in early embryos. Midplane images of RAB-5::GFP in control(RNAi), rab-5(RNAi) and rab-5 dyn-1(RNAi) embryos at pronuclear meeting, i.e., at the end of the phase of establishment of polarity. Quantitation of total cytoplasmic fluorescence intensity revealed a decrease in rab-5(RNAi) and in rab-5 dyn-1(RNAi) compared to control (p<0.001 in both cases, Student's t-test; red stars). In all panels, anterior is to the left. Scale bars, 10 µm. (JPG) [file pone.0035286.s001.jpg]

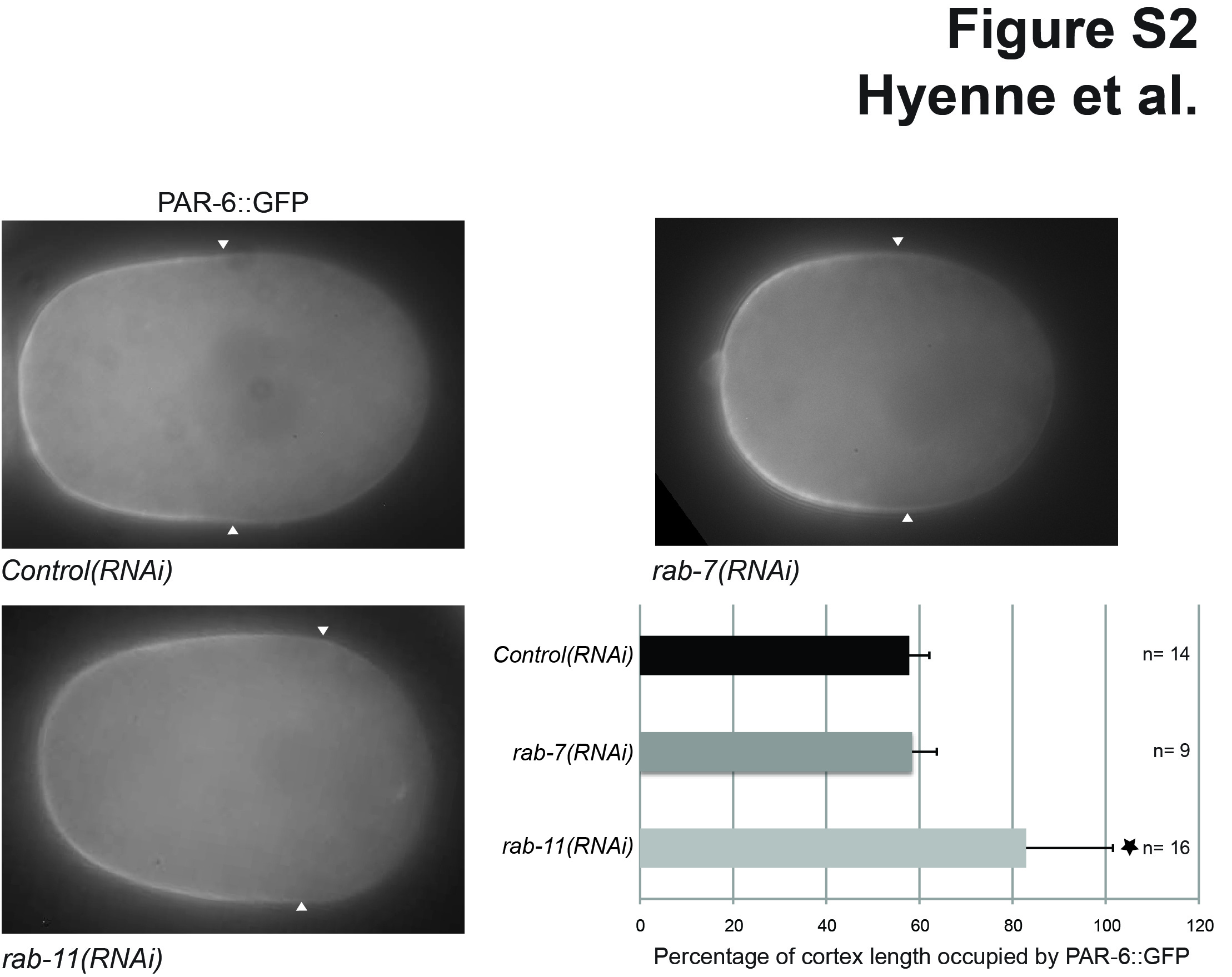

Supplement: Figure S2 — PAR-6 localization in rab-7(RNAi) and rab-11(RNAi) embryos. Midplane images of PAR-6::GFP in control(RNAi), rab-7(RNAi) and rab-11(RNAi) embryos at pronuclear meeting, i.e., at the end of the phase of establishment of polarity. Quantitation of fluorescence intensity along the cortex reveals that the size of PAR-6::GFP cortical domain is similar in control and rab-7(RNAi) (p = 0.77, Student's t-test) and enhanced in rab-11(RNAi) (p = 4.97×10^−05^, Student's t-test). In all panels, anterior is to the left. Scale bars, 10 µm. (JPG) [file pone.0035286.s002.jpg]

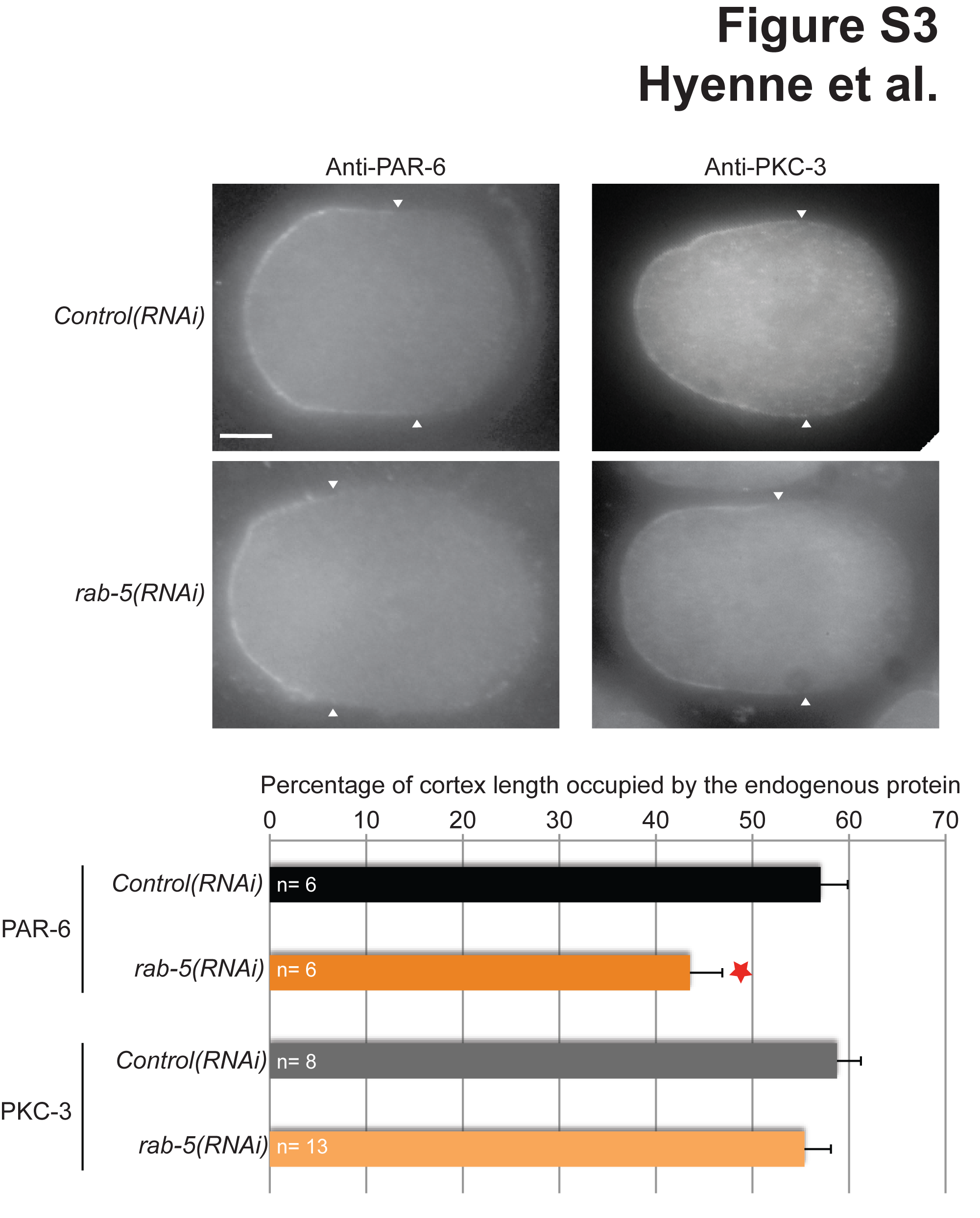

Supplement: Figure S3 — Localization of endogenous PAR-6 and PKC-3 in rab-5(RNAi) embryos. Midplane images of control(RNAi) and rab-5(RNAi) embryos at pronuclear meeting, i.e., at the end of the polarity establishment phase, labelled with anti-PAR-6 or anti-PKC-3 antibodies. Quantitation of fluorescence intensity along the cortex revealed that the size of the PAR-6 cortical domain is reduced in rab-5(RNAi) embryos (p = 0.011, Student's t-test; red star) compared to control(RNAi) embryos whereas the size of the PKC-3 cortical domain is similar in control(RNAi) and rab-5(RNAi) embryos (p = 0.41, Student's t-test). In all panels, anterior is to the left. Scale bars, 10 µm. (TIF) [file pone.0035286.s003.tif]

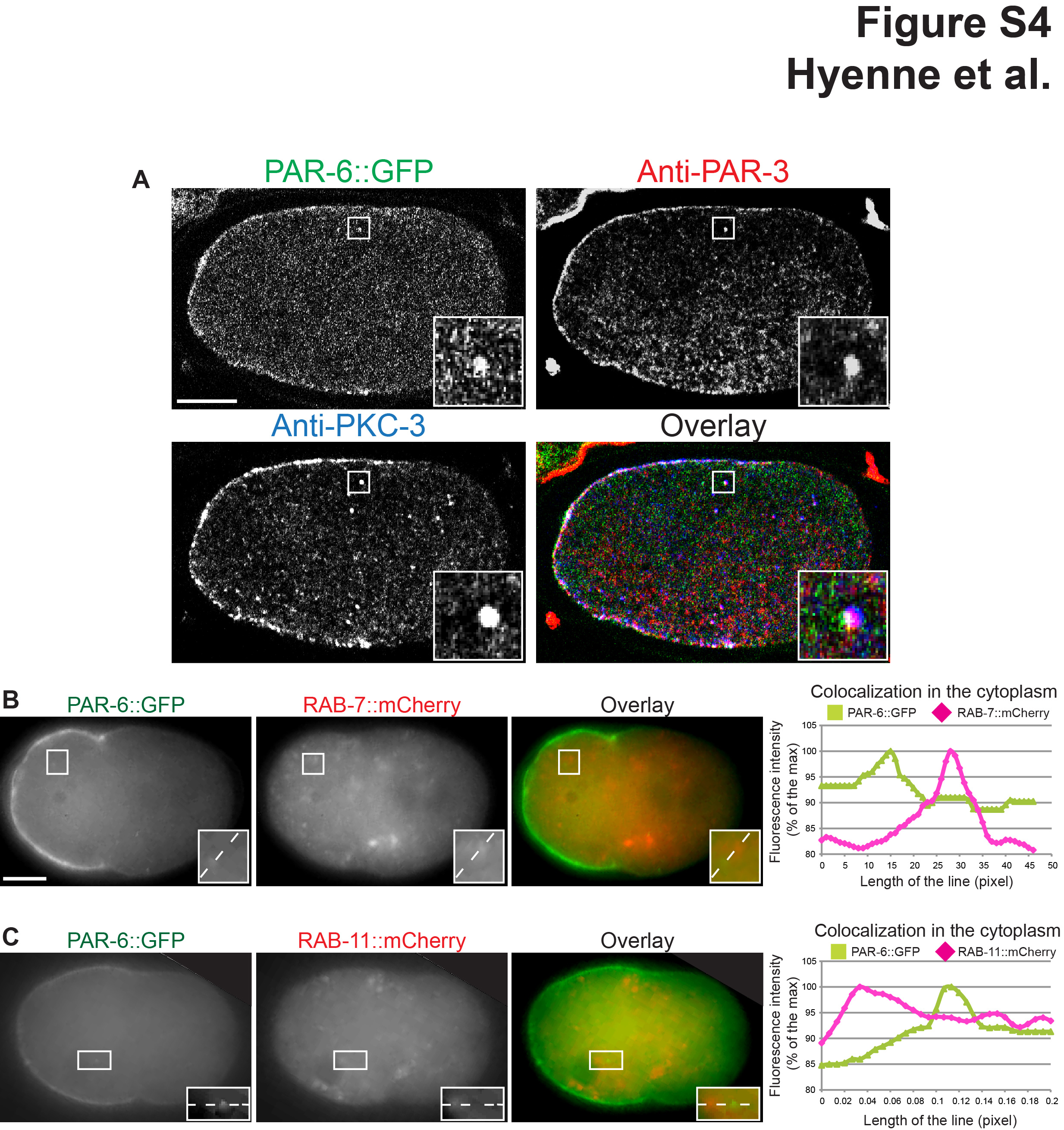

Supplement: Figure S4 — The PAR-3/PAR-6/PKC-3 complex localizes to cytoplasmic puncta. (A) Midplane confocal images of fixed embryos expressing PAR-6::GFP and labelled with anti-PAR-3 and anti-PKC-3 antibodies during the phase of establishment of polarity. The white color indicates that all three markers co-localize. Inset shows magnification of the boxed region. In all frames, anterior is to the left. Scale bar, 10 µm. (B) Midplane images of PAR-6::GFP and mCherry::RAB-7 in the cytoplasm of control embryos during the phase of establishment of polarity. The box is magnified 2.5-fold in inset. Fluorescence intensity was measured along the lines in each inset and represented for PAR-6 (green) or RAB-7 (red). No significant co-localization was observed (3.2% of co-localization, n = 53 puncta) compared to random control co-localization (3.1%, n = 31). (C) Midplane images of PAR-6::GFP and mCherry::RAB-11 in the cytoplasm of control embryos during the phase of establishment of polarity. The box is magnified 2-fold in inset. Fluorescence intensity was measured along the lines in each inset and represented for PAR-6 (green) or RAB-11 (red). No co-localization was observed (3.8% of co-localization with RAB-11 (n = 47 puncta) compared to random control co-localization (0%, n = 21). In all panels, anterior is to the left. Scale bars, 10 µm. (JPG) [file pone.0035286.s004.jpg]
