# Supplementary material for: RAB-5 Controls the Cortical Organization and Dynamics of PAR Proteins to Maintain C. elegans Early Embryonic Polarity
Source: PLoS One. 2012 Apr 24;7(4):e35286. doi: 10.1371/journal.pone.0035286 (PMC3335856; doi:10.1371/journal.pone.0035286)
Supplement: Table S1 — Strains used in this study. (DOC) [file pone.0035286.s012.doc]

**Table S1: Strains used in this study**

| Strain name | Genotype | Crossed strains | Referred to as |
| --- | --- | --- | --- |
|  | *par-2(it5ts) III.* | - | *par-2* |
| FT17 | unc-119(ed3) III; xnIs3 [par-6 : PAR-6::GFP + unc-119(+)] | - | PAR-6::GFP |
| JJ1473 | unc-119(ed3) III; zuls45 [nmy-2 : NMY-2::GFP + unc-119(+)] | - | NMY-2::GFP |
| KK866 | *itIs153[pMW1.03 GFP:PAR-2; pRF4]* | - | GFP::PAR-2 |
| UM136 | unc-119(ed3) III; xnIs3 [par-6 : PAR-6::GFP + unc-119(+)]  pwIs509[Ppie-1:mCherry:RAB-7, unc-119(+)] | FT17 and  RT1332 | PAR-6::GFP  mCherry::RAB-7 |
| UM137 | unc-119(ed3) III; xnIs3 [par-6 : PAR-6::GFP + unc-119(+)]  pwIs403[Ppie-1:mCherry:RAB-5, unc-119(+)] | FT17 and  RT1043 | PAR-6::GFP  mCherry::RAB-5 |
| UM162 | unc-119(ed3) III; xnIs3 [par-6 : PAR-6::GFP + unc-119(+)]  pwIs476[Ppie-1:mCherry:RAB-11.1, unc-119(+)] | FT17 and  RT1196 | PAR-6::GFP  mCherry::RAB-11 |
| UM177 | unc-119(ed3); pwIs47[Ppie-1:GFP:CHC-1, unc-119(+)]  PAR-6::mCherry | TH120 and  RT237 | PAR-6::mCherry  GFP::CHC-1 |
| OD183 | nnIs1[Ppie-1::gfp::moesin, unc-119(+)] | - | Dmoe::GFP |
| UM174 | nnIs1[Ppie-1::gfp::moesin, unc-119(+)]  PAR-6::mCherry | TH120 and OD183 | PAR-6::mCherry  dMoe::GFP |
